# Chemical and magnetic functionalization of graphene oxide as a route to enhance its biocompatibility

**DOI:** 10.1186/1556-276X-9-656

**Published:** 2014-12-04

**Authors:** Karolina Urbas, Malgorzata Aleksandrzak, Magdalena Jedrzejczak, Malgorzata Jedrzejczak, Rafal Rakoczy, Xuecheng Chen, Ewa Mijowska

**Affiliations:** 1 Institute of Chemical and Environment Engineering, West Pomeranian University of Technology, Szczecin, Piastow Avenue 45, Szczecin 70-311, Poland; 2 Laboratory of Cytogenetics, West Pomeranian University of Technology, Szczecin, Judyma 6, Szczecin 71-466, Poland; 3 Institute of Chemical Engineering and Environmental Protection Process, West Pomeranian University of Technology, Szczecin, Piastow Avenue 42, Szczecin 71-065, Poland

**Keywords:** Magnetic nanoparticles, Graphene oxide, Biocompatibility

## Abstract

The novel approach for deposition of iron oxide nanoparticles with narrow size distribution supported on different sized graphene oxide was reported. Two different samples with different size distributions of graphene oxide (0.5 to 7 μm and 1 to 3 μm) were selectively prepared, and the influence of the flake size distribution on the mitochondrial activity of L929 with WST1 assay *in vitro* study was also evaluated. Little reduction of mitochondrial activity of the GO-Fe_3_O_4_ samples with broader size distribution (0.5 to 7 μm) was observed. The pristine GO samples (0.5 to 7 μm) in the highest concentrations reduced the mitochondrial activity significantly. For GO-Fe_3_O_4_ samples with narrower size distribution, the best biocompatibility was noticed at concentration 12.5 μg/mL. The highest reduction of cell viability was noted at a dose 100 μg/mL for GO (1 to 3 μm). It is worth noting that the chemical functionalization of GO and Fe_3_O_4_ is a way to enhance the biocompatibility and makes the system independent of the size distribution of graphene oxide.

## Background

In recent years, graphene, well-defined 2D honeycomb-like network of carbon atoms, has attracted growing interest owing to its unprecedented combination of unique electrical, thermal, optical, and mechanical properties [1-6].

Graphene derivative, graphene oxide chemically exfoliated from oxidized graphite, is considered as a promising material for biological applications due to its surface functionalizability, amphiphilicity, and excellent aqueous processability. These extraordinary properties are mainly derived from its chemical structures composed of sp^3^ carbon domains surrounding sp^2^ carbon domain and a wide range of functional groups such as epoxy, hydroxyl, and carboxyl groups [7-10]. The chemical structure of graphene oxide and large specific surface area enable various chemical modification or functionalization and make graphene oxide an excellent platform for loading magnetic nanoparticles [11].

Magnetic nanoparticles possessing tailored surface properties and appropriate physicochemistry have been widely investigated for various applications such as hyperthermia, magnetic resonance imaging (MRI), tissue repair, drug delivery, biosensing, and bioanalysis [12-23]. In particular, the magnetite, Fe_3_O_4_, has attracted significant attention in the field of biotechnology and medicine because of its strong magnetic properties and low toxicity [24-26]. The properties of nanocrystals strongly depend on the dimension of the nanocrystals; therefore, the control of monodispersed size of nanocrystals plays an important role. Magnetic nanoparticles for the use in biomedical applications are desired to exhibit superparamagnetic properties. The superparamagnetic nature implies that the particles will not be attracted to each other, and so the risk of agglomeration in a medical setting is minimized. Magnetite is traditionally ferromagnetic in nature. However, as the size decreases to 30 nm or smaller, it loses their permanent magnetism and becomes superparamagnetic [27]. Safety concerns could ultimately prevent the adoption of magnetic nanoparticles in medicine. *In vitro* and *in vivo* toxicity results often contradict each other hence are an area that needs more research.

Recently, graphene-based materials were extensively investigated for application in biosensing [28-32], imaging [33,34], and drug delivery [35-38] as vehicles for drugs and as high-performance electrode material for capacitive deionization [39,40]. Cong et al. report on fabrication of reduced graphene oxide decorated with Fe_3_O_4_ nanoparticles through a high-temperature decomposition method [41]. This system could be used as magnetic resonance contrast agent. Shen et al. demonstrated one-step synthesis of GO-Fe_3_O_4_ nanoparticle hybrid [42]. He and Gao presented scalable, green, efficient, controllable method of preparation of superparamagnetic, processable, and conductive graphene nanosheets coated with magnetite nanoparticles [43]. He et al. showed attachment of magnetite nanoparticles to GO surface with covalent bonding [44]. Yang et al. described GO-Fe_3_O_4_ nanoparticle hybrid supporting doxorubicin hydrochloride (anticancer drug) [35]. This system could be easily removed from water by an external magnetic field. Zheng and Li reported on fabrication of a magnetite nanoparticle-decorated graphene oxide (Fe_3_O_4_-GO) and reduced graphene oxide (Fe_3_O_4_-rGO) loaded with β-lapachone (anticancer drug), *in vitro* anticancer efficacy and cytotoxicity of obtained materials [45]. Bai et al. presented results of study on the inductive heating property of graphene oxide sheets decorated with magnetite nanoparticles in AC magnetic field [46]. The potential of the obtained nanocomposite was evaluated for localized hyperthermia treatment of cancer cells.

Herein, we present new facile approach for production of the monodispersed Fe_3_O_4_ nanoparticles and magnetic attachment of magnetite nanoparticles to graphene oxide sheets with different flake size distributions. The mean size of the obtained magnetite nanoparticles is about 8 nm. Additionally, we performed cytocompatibility study on the influence of these molecular hybrids on the mitochondrial activity of L929 cell line with WST1 assay in respect to the GO and pristine iron oxide nanoparticles. The cellular response was verified with different concentration (0.0, 3.125, 6.25, 12.5, 25.0, 50.0, 100.0 μg/mL) of the nanomaterials.

## Methods

### Preparation of graphene oxide-Fe_3_O_4_ nanoparticle hybrid

#### Synthesis of graphene oxide

Two types of samples of graphene oxide were synthesized by oxidation of graphite with various size of flakes (with narrow and broad size distribution) using the modified Hummer's method fully described elsewhere [47]. To a mixture of 6 g KMnO_4_ and 1 g graphite, 120 mL of concentrated sulfuric acid and 15 mL of orthophosphoric acid were poured. It was heated to 50°C and stirred for 24 h. The resulting mixture was added to ice (150 mL) with 1 mL of H_2_O_2_ (30%) and centrifuged. The separated solid product was washed two times with water and 30% HCl and ethanol and left for vacuum drying for 12 h at 70°C. The sample with broad size distribution is named B-GO, and the sample with narrow size distribution is named N-GO.

#### Synthesis of magnetite nanoparticles

Magnetic Fe_3_O_4_ nanoparticles were synthesized by co-precipitation of Fe^2+^ and Fe^3+^ aqueous salt solutions using NH_3_ · H_2_O as the precipitating agent in order to adjust the pH value. It should be noticed that the size, shape, and composition of nanoparticles may be controlled by means of the type of salts used, Fe^2+^ and Fe^3+^ ratio, pH, and ionic strength of the media. To synthesize Fe_3_O_4_ nanoparticles, the solutions of 3.9 g Mohr's salt (NH_4_)_2_Fe(SO_4_)_2_ · 6H_2_O in 100 mL H_2_O (0.1 M) and 4.8 g NH_4_Fe(SO_4_)_2_ · 12H_2_O in 100 mL H_2_O (0.1 M) were prepared and mixed with a molar ratio of 1:2. Ammonia aqueous solution was dropped into the mixture slowly until the pH value of the solution reached 9. The complete precipitation of Fe_3_O_4_ was expected between pH 9 and 14. The overall reaction may be written as follows:

$$\begin{array}{l} {\left({\text{NH}}_{4}\right)}_{2}\text{Fe}{\left({\text{SO}}_{4}\right)}_{2}\cdot 6H_{2}O\;+\;2\;{\text{NH}}_{4}\text{Fe}{\left({\text{SO}}_{4}\right)}_{2}\cdot 12H_{2}O\\ \;+\;8\;{\text{NH}}_{4}\text{OH}\;\to \\ \;\to \;{\text{Fe}}_{3}O_{4}\cdot 4H_{2}O\;+\;6\;{\left({\text{NH}}_{4}\right)}_{2}{\text{SO}}_{4}+\;14\;H_{2}O \end{array}$$

The magnetite synthesis route was carried out under the action of a rotating magnetic field (RMF). A liquid-filled glass container was placed inside the three-phase stator of an induction squirrel-cage motor which generated the RMF. This kind of the magnetic field might be used to augment the process intensity instead of a mechanical mixing. One of the advantages of the RMF is the possibility to apply it to generation and control of the hydrodynamic states for the magnetic particle mixing systems. In the experimental procedure, the frequency of the RMF was equal to 50 Hz. The intensity of the magnetic field could be 25 mT. The more detailed information about the experimental setup and the measurements of the magnetic field for the tested apparatus may be found here [48]. Finally, the precipitate was collected by filtration and washed three times with deionized water and then dried.

#### Synthesis of graphene oxide-Fe_3_O_4_ nanoparticle hybrid

**Figure 1:**
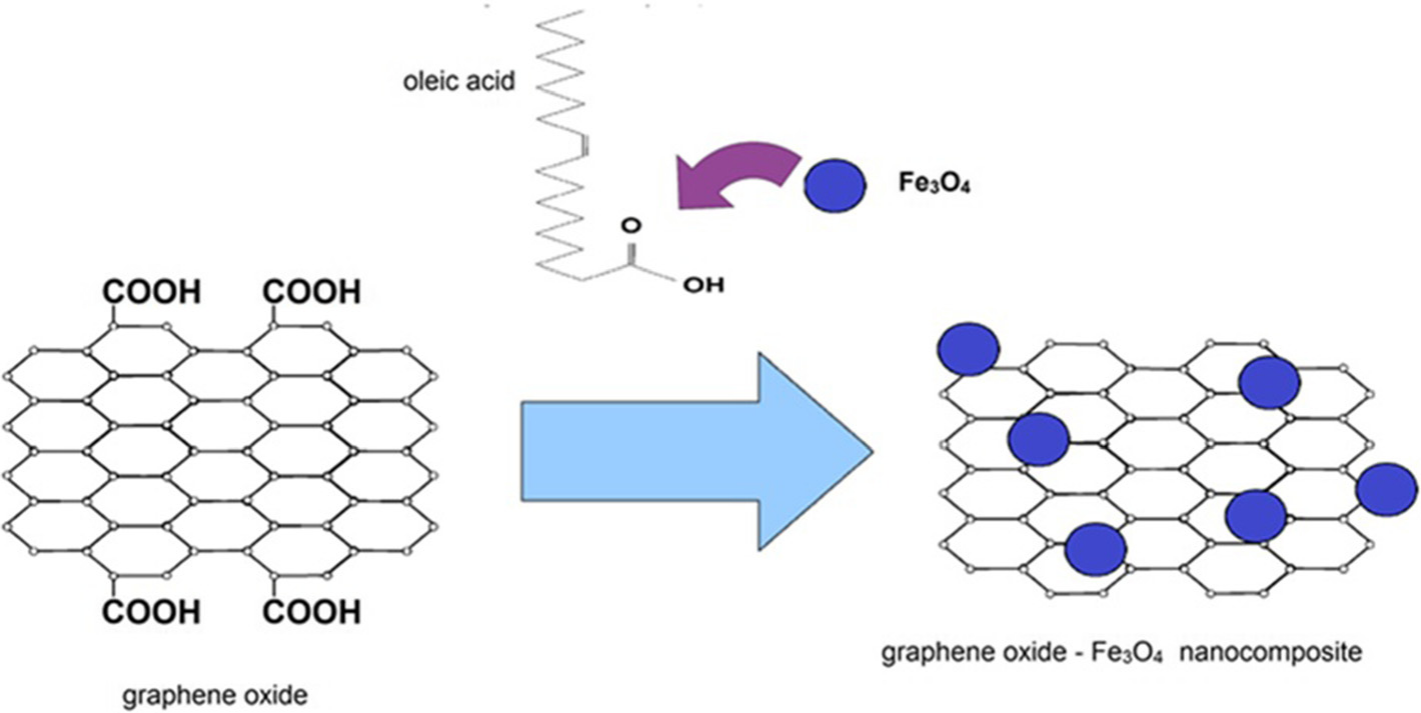
**Schematic of the synthesis of GO-Fe**_**3**_**O**_**4**_**nanoparticle hybrid.**

The synthesis process of GO-Fe_3_O_4_ nanocomposite is schematically illustrated in Figure 1. Twenty milligrams of each of graphene oxide sample (B-GO and N-GO) was exfoliated in 60 mL H_2_O by ultrasonication to produce a homogeneous graphene oxide water-based suspension. Then, the carboxylic groups on the graphene oxide surface were activated with 8 mg of *N*-hydroxysuccinimide (NHS) and 10 mg of 1-(3-dimethylaminopropyl)-3-ethylcarbodiimide (EDC). The surface of iron oxide (20 mg) was modified with oleic acid (3 mL) and then stirred and sonicated for 1 h. The mixture of modified iron oxide and graphene oxide was stirred for 48 h. In the next step, the mixture was filtered by a polycarbonate membrane and washed several times with water and ethanol. Finally, the obtained product was dried for 12 h at 100°C. The sample with broad size distribution of graphene oxide flakes is named B-GO-Fe_3_O_4_, and the sample with narrow size distribution is named N-GO-Fe_3_O_4_.

### Characterization

High-resolution transmission electron microscopy (HRTEM) (FEI Tecnai F30, Frequency Electronics Inc., Mitchel Field, NY, USA) was employed to examine the morphology of the samples and the size and distribution of the magnetite nanoparticles. X-ray diffraction technique (X-ray diffractometer Philips X'Pert PRO, PANalytical B.V., Almelo, The Netherlands, K_*α*1_ = 1.54056 Å) was used to investigate the structure of the samples and to estimate the average size of magnetite nanoparticles. In order to study the thickness of obtained graphene oxide flakes and nanocomposite, atomic force microscopy (Nanoscope V Multimode 8, Bruker AXS, Mannheim, Germany) was employed. IR absorption spectra were collected on the Nicolet 6700 FTIR spectrometer (Thermo Nicolet Corp., Madison, WI, USA). In order to investigate the thermal behavior of the samples, thermogravimetric analysis was performed on the SDT Q600 simultaneous TGA/DSC (TA Instruments Inc., Milford, MA, USA) under an air flow of 100 mL/min at heating rate of 5°C/min. Raman spectra were acquired on the inVia Raman Microscope (Renishaw PLC, New Mills Wotton-under-Edge, Gloucestershire, UK) at an excitation wavelength of 785 nm.

#### Cell culture

The cell line of mouse fibroblasts (L929) were seeded on the 96-well plates at the density of 7.4 × 10^3^ per well. Cells were maintained using DMEM cell culture medium (Gibco Corp., Grand Island, NY, USA) supplemented with 10% heat-inactivated fetal bovine serum (Gibco Corp., Grand Island, NY, USA), 0.4% streptomycin/penicillin (Sigma-Aldrich Corp., St. Louis, MO, USA), and 2 mM L-glutamine (Sigma-Aldrich Corp., St. Louis, MO, USA) at 37°C, 5% CO_2_, and 95% humidity. The 200 μL/well final volume of culture medium was used in experiment.

#### The cytocompatibility study

The cytocompatibility of nanomaterials was tested using WST-1 Cell Proliferation Assay (Roche Applied Science, Penzberg, Germany) [49]. The test principle is based on the transformation of WST-1 salt [2-(4-iodophenyl)-3-(4-nitrophenyl)-5-(2,4-disulfophenyl)-2H-tetrazolium] into water-soluble colored formazan by mitochondrial dehydrogenases [50] that are active in rapidly dividing cells [51]. The generation of the dark yellow colored formazan is directly correlated to the number of the metabolically active cells; therefore, the cell number can be quantified by the photometric detection of the formazan. There are several similar proliferation assays using other tetrazolium salts, such as MTT [3-(4,5-dimethylthiazol-2-yl)-2,5-diphenyltetrazolium bromide], XTT [2,3-bis(2-methoxy-4-nitro-5-sulfophenyl)-2H-tetrazolium-5-carboxanilide], and MTS [3-(4,5-dimethylthiazol-2-yl)-5-(3-carboxymethoxyphenyl)-2-(4-sulfophenyl)-2H-tetrazolium] available on the market. The main advantage of WST-1 test over those mentioned above is the solubility of reduced WST-1 salt. It also requires no washing, harvesting, or solubilization of cells. To perform the assay, L929 cells were plated in the 96-well plates for 24 h. After incubation period from cells seeding, N-GO, B-GO, N-GO-Fe_3_O_4_, B-GO-Fe_3_O_4_, and Fe_3_O_4_ were introduced separately to cells with different final concentrations (0.0, 3.125, 6.25, 12.5, 25.0, 50.0, 100.0 μg/mL) in culture medium. Cells were incubated with nanomaterial for 24 h. Cells maintained in prepared medium without adding tested samples were taken as a control. To each well, 20 μL of WST-1 solution was added and incubated for additional 30 min at 37°C. After incubation, the absorbance at 450 nm, according to manufacturer's instructions, was recorded on the Sunrise Absorbance Reader (Tecan Group Ltd., Männedorf, Switzerland). All of the experiments were conducted in triplicate.

#### Statistical analysis

All experiments were repeated at least three times. The results are given in the form: mean values ± standard deviation (SD). All results were compared using Student's *t*-test. Differences are considered significant at a level of *p* < 0.05.

## Results and discussion

**Figure 2:**
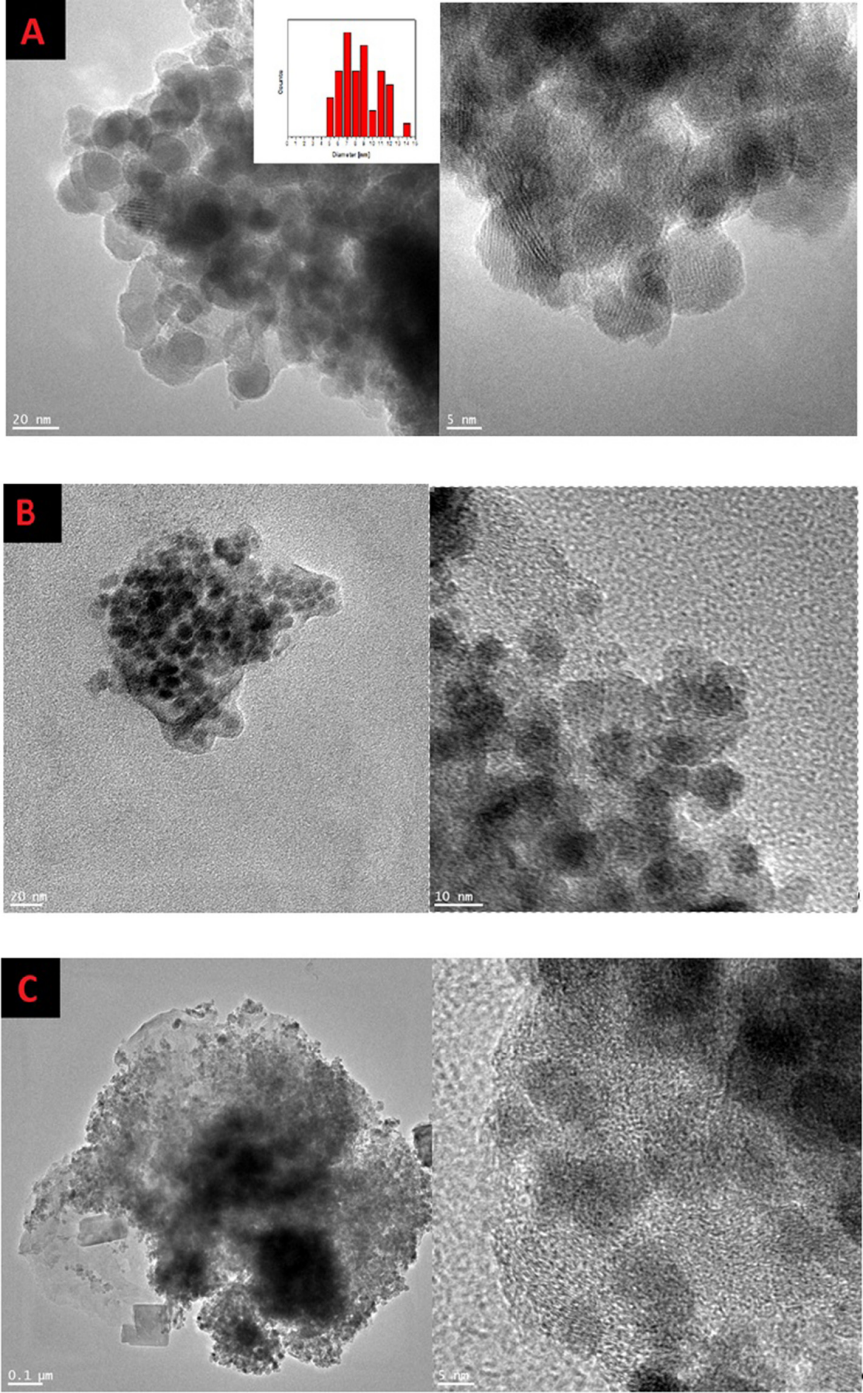
**TEM images of Fe**_**3**_**O**_**4**_**with diameter size distribution (A), B-GO-Fe**_**3**_**O**_**4**_**(B), and N-GO-Fe**_**3**_**O**_**4**_**(C).**

Transmission electron microscopy (TEM) was used for characterization of starting materials and final products. Representative TEM images of Fe_3_O_4_, B-GO-Fe_3_O_4_, and N-GO-Fe_3_O_4_ nanoparticle hybrid are shown in Figure 2. Images of magnetite indicate a spherical shape of magnetite nanoparticles. The histogram presenting diameter distribution of the Fe_3_O_4_ nanoparticles is placed as an inset of Figure 2A. The diameter of the nanoparticles is in the range of 5 to 14 nm with strong peak at 8 nm. Uniform coverage of graphene oxide with magnetite nanoparticles can be observed in the images of the obtained nanocomposites (Figure 2B,C).

**Figure 3:**
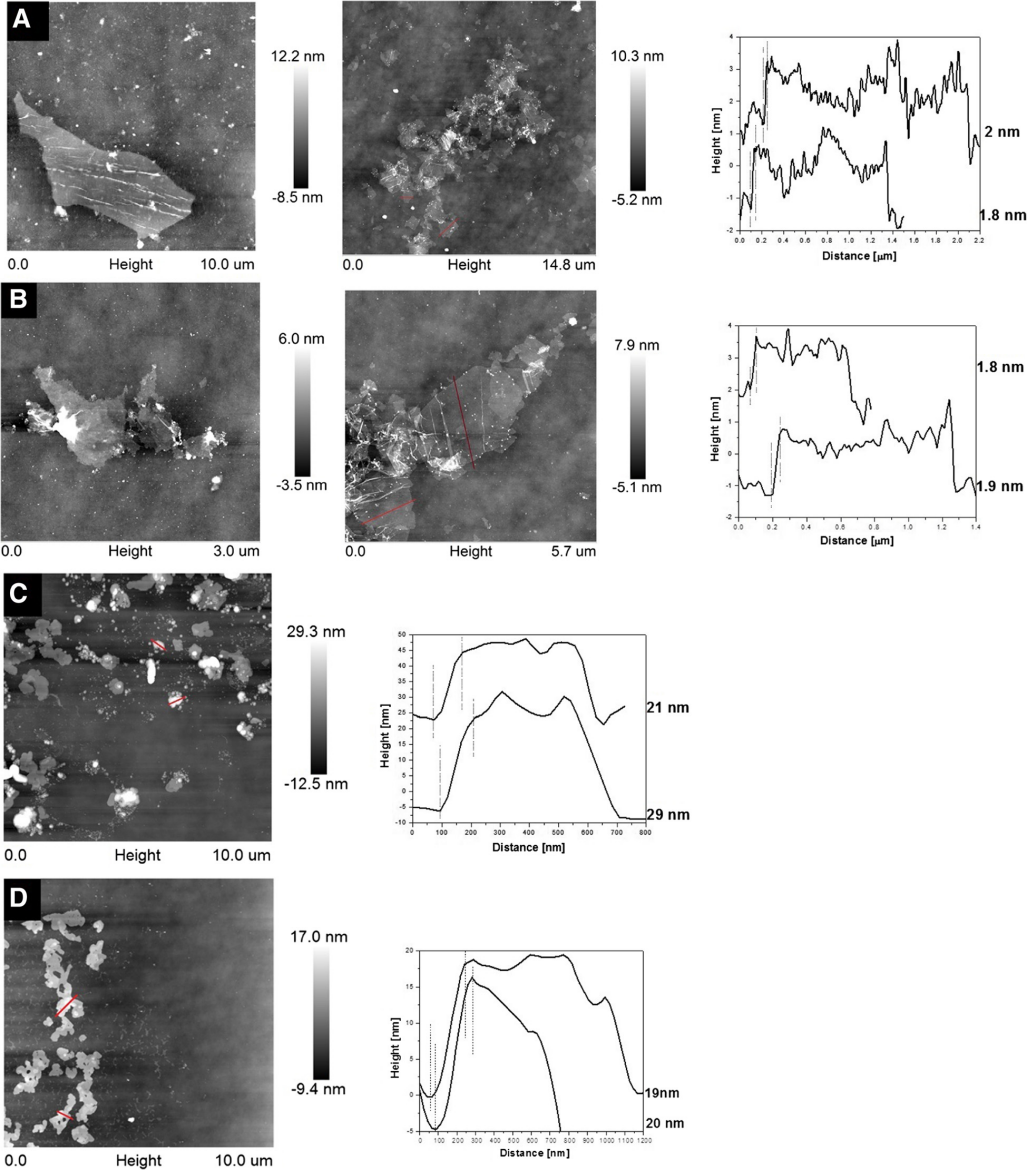
**AFM images and height profiles of B-GO (A), N-GO (B), B-GO-Fe**_**3**_**O**_**4**_**(C), and N-GO-Fe**_**3**_**O**_**4**_**(D).**

**Figure 4:**
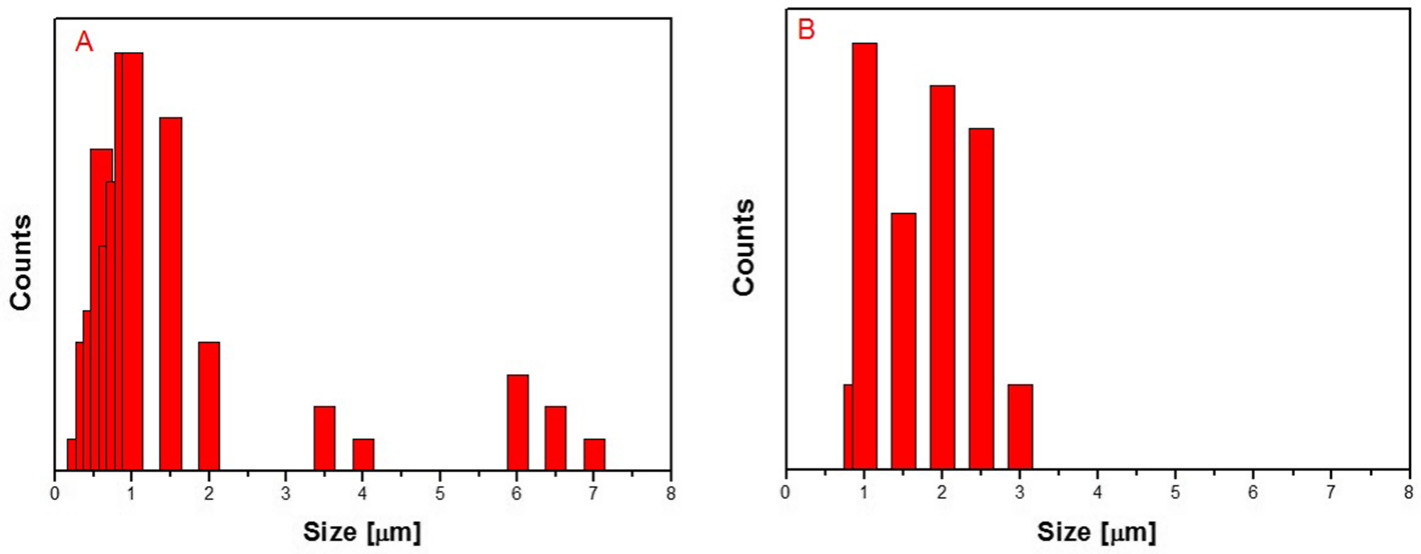
The size distributions of graphene oxide flakes in B-GO (A) and N-GO (B).

To examine the morphology of the samples and the thickness of graphene oxide flakes, atomic force microscopy was employed. Atomic force microscopy (AFM) results of B-GO, N-GO, and B-GO-Fe_3_O_4_ and N-GO-Fe_3_O_4_ nanoparticle hybrid are presented in Figure 3. They indicate the change in the thickness of the flakes before and after functionalization - from 2 to 18 to 30 nm in both kinds of hybrids due to the deposition of the nanoparticles on graphene oxide flakes.AFM characterization is a useful tool for estimating the size distribution of the GO flakes. Figure 4A presents broad size distribution of graphene oxide flakes (from 0.5 to 7 μm) whereas Figure 4B presents narrower size distribution (from 1 to 3 μm).

**Figure 5:**
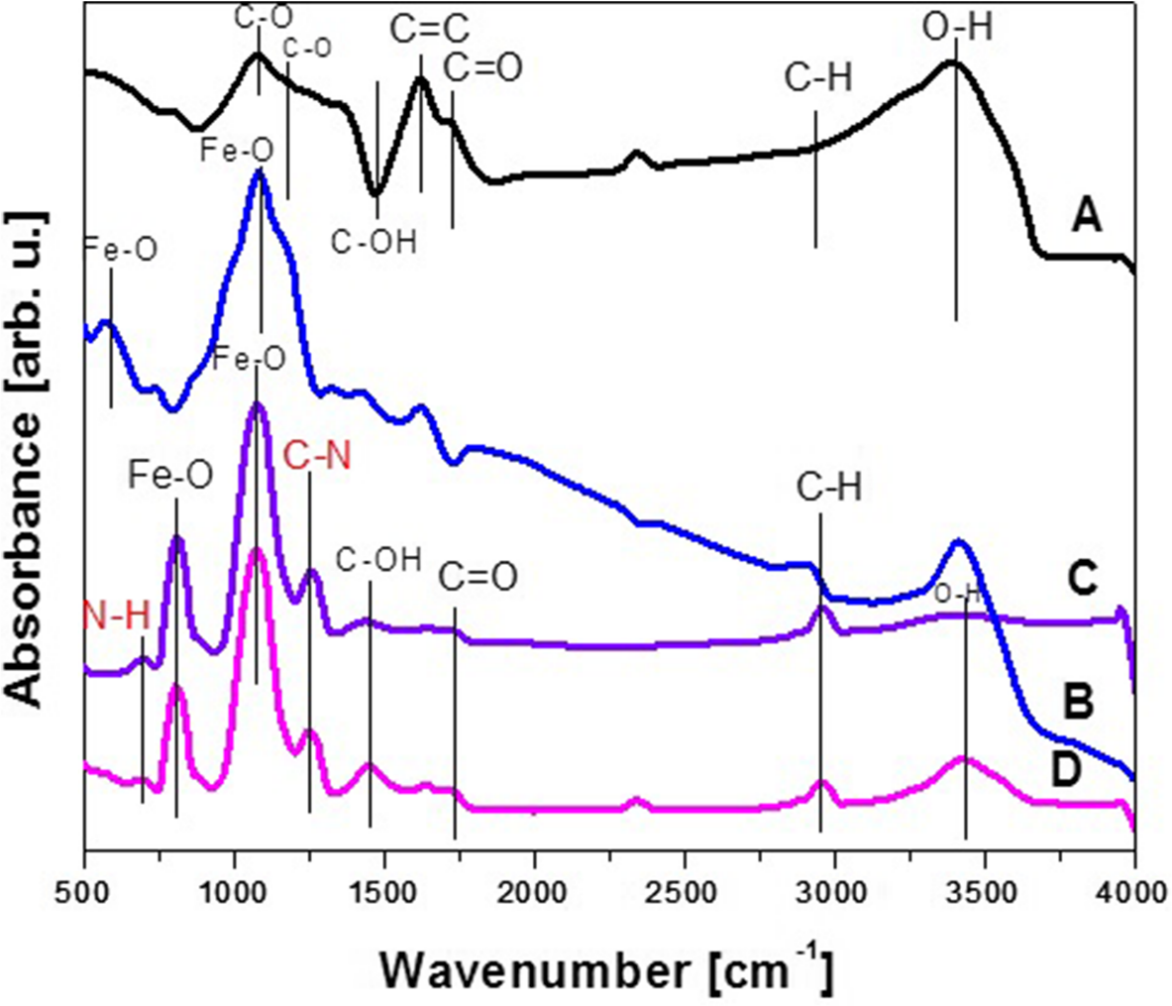
**FTIR spectra of GO (A), Fe**_**3**_**O**_**4**_**(B), B-GO-Fe**_**3**_**O**_**4**_**(C), and N-GO-Fe**_**3**_**O**_**4**_**(D).**

In order to follow the efficiency of the covalent functionalization of graphene oxide and iron oxide nanoparticle, the Fourier transform infrared (FTIR) spectroscopy was employed. Figure 5 presents FTIR spectra of GO, Fe_3_O_4_, B-GO-Fe_3_O_4_, and N-GO-Fe_3_O_4_. Figure 5A shows spectrum of GO with following peaks: 1,078 cm^-1^ corresponding to C-O stretching vibration mode related to the presence of the alkoxy group, 1,180 cm^-1^ attributed to C-O from the epoxy group, 1,475 cm^-1^ from C-OH carboxyl group, 1,625 cm^-1^ associated with the presence of C = C bond, and 1,742 cm^-1^ assigned to C = O stretching vibration mode in carboxyl group. Peak at 2,952 cm^-1^ originates from C-H bond. This spectrum confirms the presence of alkoxy, epoxy, and carboxyl group characteristic to graphene oxide. Figure 5B depicts FTIR absorption spectrum of Fe_3_O_4_ dominated by peaks at 570 and 1,059 cm^-1^ assigned to the presence of Fe-O bonds typical to magnetite. In the spectra of B-GO-Fe_3_O_4_ and N-GO-Fe_3_O_4_ (Figure 5C,D, respectively) peak from Fe-O bond has shifted from 570 to 809 cm^-1^ after functionalization. New peaks at 698 and 1,259 cm^-1^ occurred. The first one is attributed to N-H bond, and the second one is corresponded to C-N bond. Therefore, the presented spectra provide the proof for successful functionalization of GO with Fe_3_O_4_ via linkage of the oleic acid surrounding the iron oxide and NHS bonded to GO. The peak originating from the functional groups of graphene oxide are also present in the spectra of B-GO-Fe_3_O_4_ and N-GO-Fe_3_O_4_ which means that the starting material (GO) did not undergo the reduction during the functionalization.

**Figure 6:**
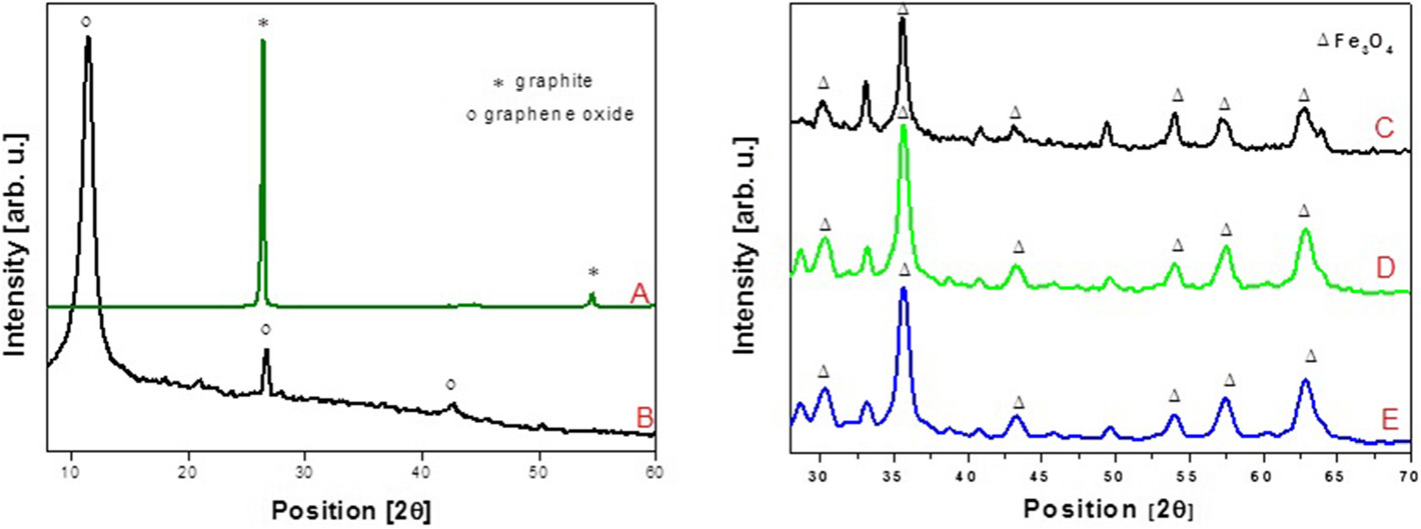
**XRD patterns of graphite (A), GO (B), Fe**_**3**_**O**_**4**_**(C), B-GO-Fe**_**3**_**O**_**4**_**(D), and N-GO-Fe**_**3**_**O**_**4**_**(E).**

X-ray diffraction (XRD) patterns of graphite, graphene oxide, B-GO-Fe_3_O_4_, and N-GO-Fe_3_O_4_ hybrid are shown in Figure 6. XRD spectrum of graphite is dominated by intense and narrow peak at 2θ = 26.48^o^ corresponding to reflection in (002) planes of well-ordered graphene layers. The lack of this reflection in the diffractogram of graphene oxide and the difference in the layer distances of the starting material and the final product confirm the completion of the oxidation reaction. Positions of the peaks and their relative intensities shown in XRD pattern of Fe_3_O_4_, B-GO-Fe_3_O_4_, and N-GO-Fe_3_O_4_ nanoparticle hybrid (Figure 6C,D,E) are consistent with the standard XRD data for magnetite (ICSD 65339). The average size of magnetite nanoparticles, calculated from the Scherrer's equation [52], is about 8 nm, which is consistent with the from TEM observations.

**Figure 7:**
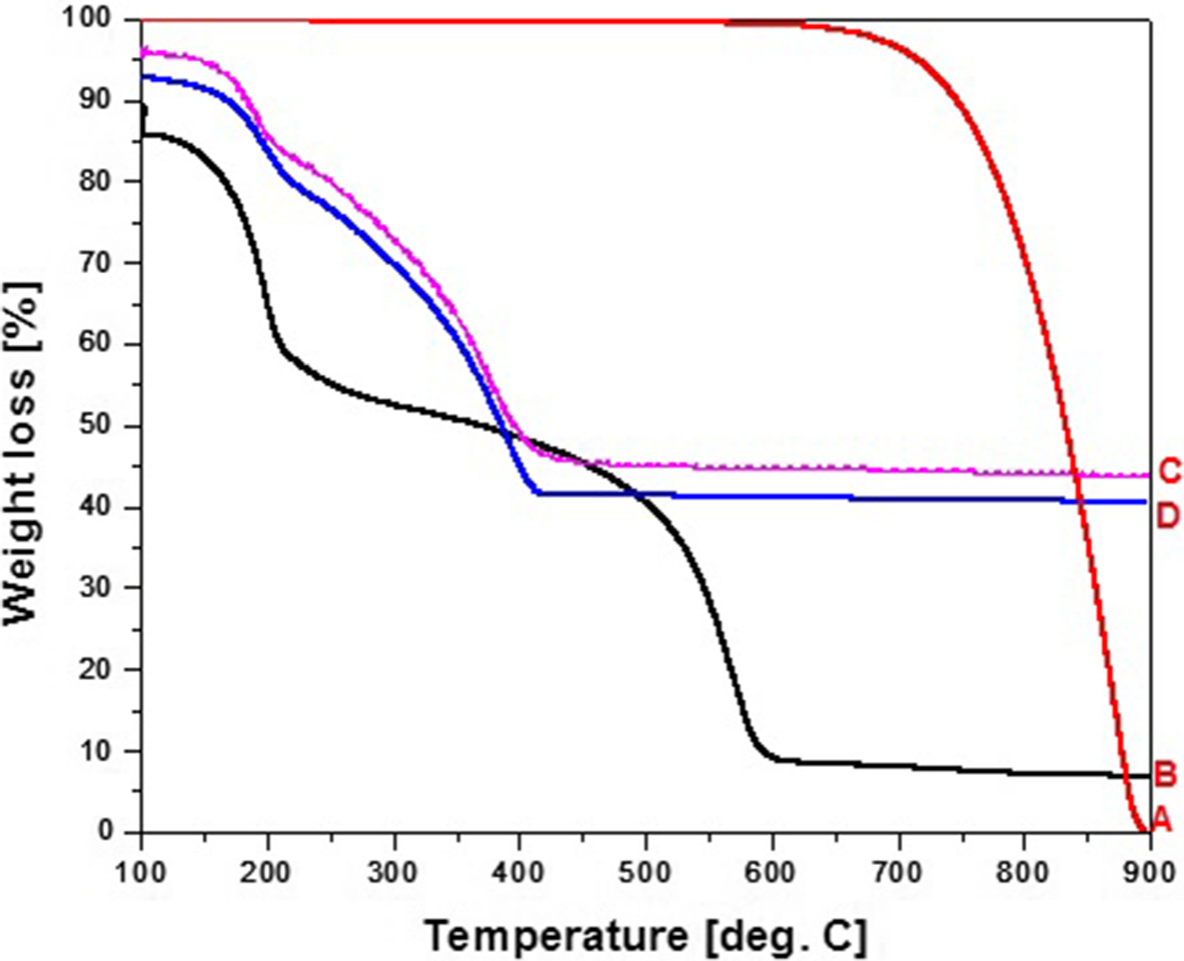
**TG curves of graphite (A), GO (B), B-GO-Fe**_**3**_**O**_**4**_**(C), and N-GO-Fe**_**3**_**O**_**4**_**(D).**

Thermal gravimetric analysis is a useful tool to determine the composition of the samples. Figure 7 presents thermogravimetric (TG) curves of graphite, GO and B-GO-Fe_3_O_4_ and N-GO-Fe_3_O_4_ heated in the air_._ Weight loss observed at 100°C is associated with the decomposition of physically adsorbed water. In graphene oxide (Figure 7B), the next weight loss occurred between 150°C and 300°C and it can be assigned to the decomposition and evaporization of oxygen-containing functional groups. After reaching 500°C (in graphite at 700°C - Figure 7A), the carbon skeleton underwent bulk pyrolysis. TG curves of nanocomposites (Figure 7C,D) indicate that the amount of loaded Fe_3_O_4_ is approximately 43 wt.% for B-GO-Fe_3_O_4_ and 40% for N-GO-Fe_3_O_4_. Both curves show the weight loss between 150°C and 300°C which means that graphene oxide did not underwent the reduction during the deposition of magnetite nanoparticles.

**Figure 8:**
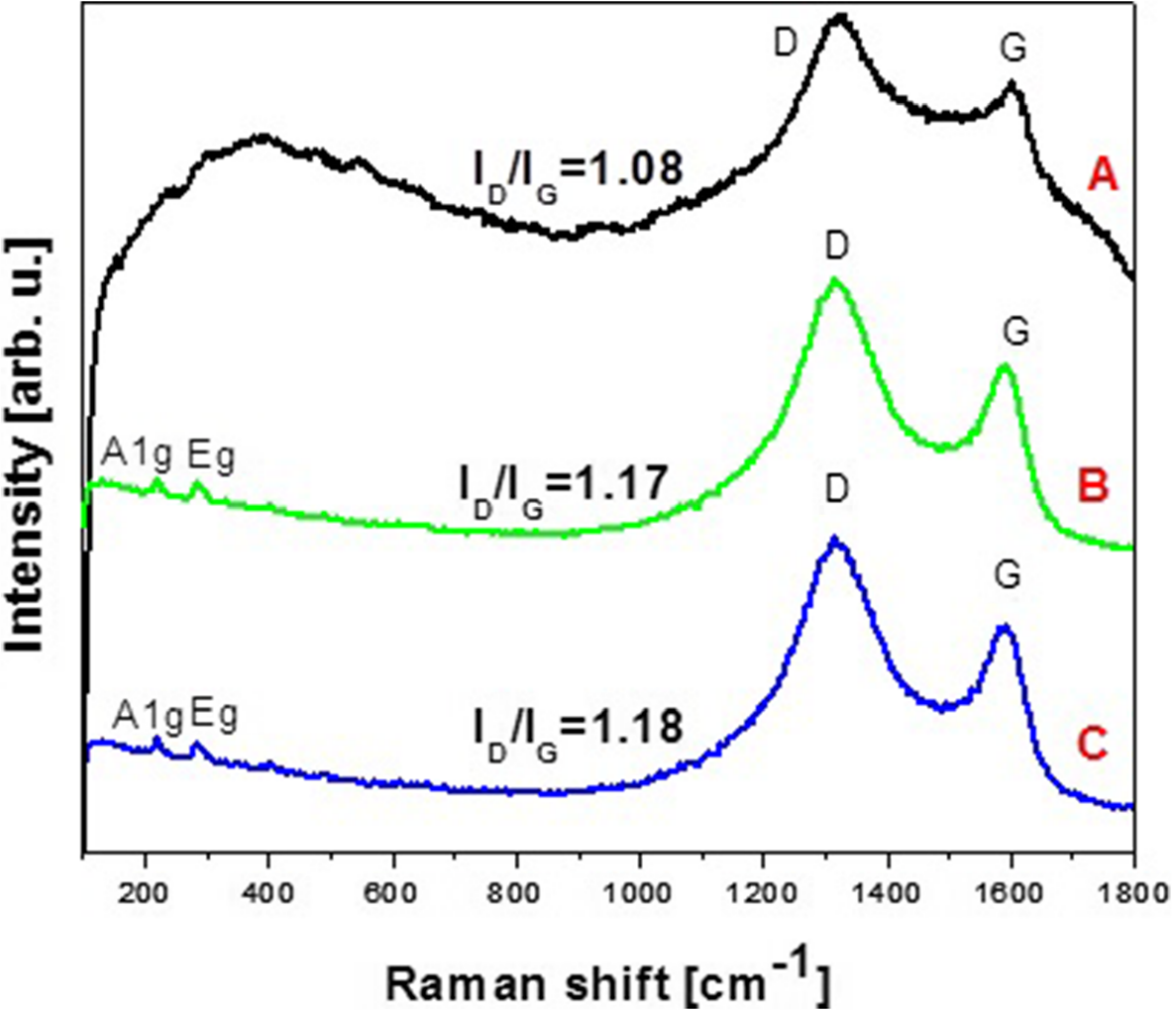
**Raman spectra of GO (A), B-GO-Fe**_**3**_**O**_**4**_**(B), and N-GO-Fe**_**3**_**O**_**4**_**(C).**

Raman spectroscopy is a powerful nondestructive tool to characterize carbonaceous materials, particularly for distinguishing ordered and disordered crystal structures of carbon. The significant structural changes occurring during the functionalization of GO are also reflected in their Raman spectra. Figure 8 presents Raman spectra of graphene oxide, B-GO-Fe_3_O_4_ and N-GO-Fe_3_O_4_. All spectra exhibit peaks at 1,318 and 1,602 cm^-1^ corresponded to D and G bands, respectively. The D band is associated with a certain fraction of sp^3^ carbon atoms obtained due to an amorphization of graphite during the oxidation process, whereas the G mode originates from the in-plane vibration of sp^2^ carbon atoms [53,54]. The ratio of *I*_*D*_/*I*_*G*_ increases after functionalization of graphene oxide with magnetite nanoparticles in the case of both hybrid systems indicating successful functionalization of the starting material. Due to the presence of Fe_3_O_4_ nanoparticles in nanocomposites in the spectra of B-GO-Fe_3_O_4_ (Figure 8B) and N-GO-Fe_3_O_4_ (Figure 8C) additional peaks in the range of 200 to 300 cm^-1^ can be observed.

**Figure 9:**
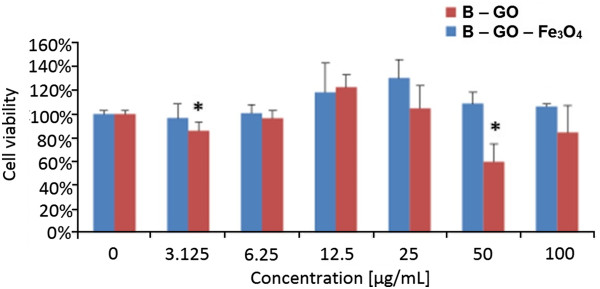
**Relative viability of fibroblast cell line L929 exposed to B-GO and B-GO-Fe**_**3**_**O**_**4**_**hybrid*.*** The cell viability is presented as percentage of the mean value. Bars represent standard deviation, and the symbol asterisk indicates statistically significant difference (*p* < 0.05).

The good biocompatibility and safety of nanomaterials is fundamental for its medical application. The first step of this kind of investigations is *in vitro* analyses, e.g., mitochondrial activity. Here, five different samples have been examined for their cytocompatibility with L929 mouse fibroblasts: two samples of the nanocomposites of graphene oxide-Fe_3_O_4_ nanoparticles with different size distributions of GO flakes: B-GO-Fe_3_O_4_ and N-GO-Fe_3_O_4_, two samples of reference graphene oxide (B-GO and N-GO) and Fe_3_O_4_ nanoparticles. The presented biocompatibility study shows the differences in mitochondrial activity of L929 cells that depend on the type of nanomaterial and concentration (0.0, 3.125, 6.25, 12.5, 25.0, 50.0, 100.0 μg/mL). As shown in Figure 9 (mitochondrial activity of the samples with broader size distribution), no significant cytotoxicity of the GO-Fe_3_O_4_ sample was detected for B-GO-Fe_3_O_4_. At the concentration between 3.125 and 100 μg/mL, minimal reduction of the cell mitochondrial activity was observed. Little reduction of cell viability was noted at a dose of 3.125 μg/mL in B-GO-Fe_3_O_4_ sample. In the sample with 25 μg/mL concentration, the mitochondrial activity for L929 cells was the highest. Interestingly, mitochondrial activity of the cells when interacting with the pristinegraphene oxide in the concentration between 50 and 100 μg/mL is significantly reduced. At a dose of 50 μg/mL, mitochondrial activity was reduced by approximately 40% for B-GO material. For 100 and 3.125 μg/mL concentration, cell viability was reduced to approximately 80%. Lower cytotoxicity was observed at a dose of 6.25 μg/mL in cell cultures treated with this graphene oxide. It means that the chemical functionalization of GO and deposition of Fe_3_O_4_ enhance the biocompatibility of the system.

**Figure 10:**
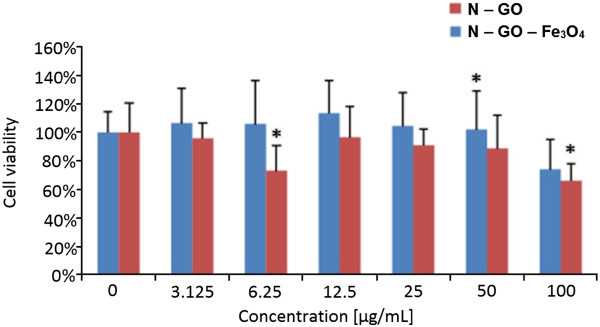
**Relative viability of fibroblast cell line L929 exposed to N-GO and N-GO-Fe**_**3**_**O**_**4**_**hybrid*.*** The cell viability is presented as percentage of the mean value. Bars represent standard deviation. Symbol asterisk indicates statistically significant difference (*p* < 0.05).

Figure 10 presents cell viability upon interaction with N-GO-Fe_3_O_4_. Here, the best biocompatibility was noticed for the samples at concentration of 12.5 μg/mL for N-GO-Fe_3_O_4_ and N-GO, respectively_._ These results are similar to that obtained for samples with broader size distribution. However, at a dose of 100 μg/mL, the highest reduction of the cell viability was noticed. Little reduction of cell viability was noted at a dose 3.125 μg/mL in N-GO-Fe3O4 and GO sample. However, it can be observed that N-GO induces higher reduction of mitochondrial activity than hybrid samples. The same trend was observed in the sample with broad flake size distribution. It proves that the nanocomposite material is more biocompatible than pristine GO platforms. Furthermore, the chemical functionalization of GO and Fe_3_O_4_ leads to enhancement of the biocompatibility of the system and its independence of the size of GO. In the other study [35], functionalized GO and GO-Fe_3_O_4_ were tested on HeLa cell line. The WST-1 assay showed differences at mitochondrial activity between GO and GO-Fe_3_O_4_. Yang et al. indicated that the GO samples presented higher cytotoxicity than GO-Fe_3_O_4_[35]. Those results are in agreement with our study.

The effects of GO on mouse fibroblast cells depend on GO dose, and as shown in the study of Wang et al. [55], the effects also depend on the culture time. The most cytotoxic effect of graphene oxide on human fibroblast cells (HDF) was observed on the fifth day of culture at the doses of 50 and 100 μg/mL. Similar results were noticed in tumor cell lines, e.g., human gastric cancer MGC803, human breast cancer MCF-7 and MDA-MB-435, and liver cancer HepG2 [55]. In our study, the effects of experimental samples on the cell culture were monitored for 24-h period, but as mentioned earlier, cell viability was reduced the most, to approximately 60%, at GO's concentration of 50 μg/mL. Chang et al. [56] using CCK-8 assay and A549 cells made observation that preparation method of GO has influence of relative cell viability. The influence of different GO samples (s-GO with smaller size, l-GO with larger size, and m-GO mix) on the mitochondria activity may vary. m-GO's effect on cell cultures was insignificant at the concentration range of 100 to 200 μg/mL. When the s-GO was tested, the cell viability was reduced the most at concentration between 50 and 200 μg/mL. It also has been noticed that the difference between some studies might come from the different sample properties and various cell lines. Incubation time can also influence the cell response [55,57]. In our study, the GO sample with concentration of 100 μg/mL demonstrated weak toxicity. We suggest that higher concentration (100 μg/mL) of graphene oxide may influence on harder GO migration to cell cytoplasm. We also found that GO material shows relatively good cytocompatibility at the concentration of 12.5 μg/mL and that result corresponds to the result obtained by Wojtoniszak et al. [58].

**Figure 11:**
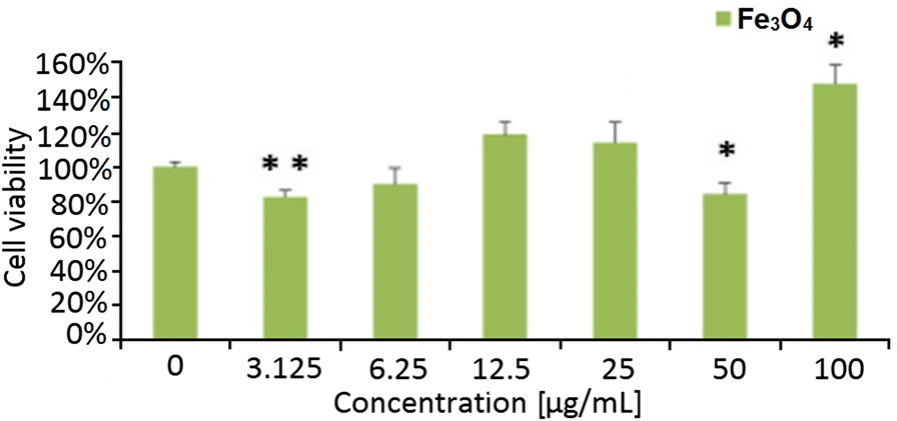
**Relative viability of fibroblast cell line L929 exposed to Fe**_**3**_**O**_**4**_**nanoparticles*.*** The cell viability is presented as percentage of the mean value. Bars represent standard deviation, and symbol asterisk indicates statistically significant difference (*p* < 0.05).

For the Fe_3_O_4_ material, lower mitochondrial activity was noticed at concentration of 3.125 and 50 μg/mL (Figure 11). In the samples of Fe_3_O_4_ with concentration of 6.25 μg/mL, the viability was reduced to 90%. At a dose of 100 μg/mL, the highest mitochondrial activity was observed for the Fe_3_O_4_ material. Analysing the results demonstrated by Shundo et al., one can see that even higher concentration of iron oxide nanoparticles, between 125 and 1,000 μg/mL, does not reduce the cell viability [59]. This can be explained by low uptake of Fe_3_O_4_ samples by the cells. Our study suggests that through the deposition of iron oxide nanoparticles via covalent linkage on the graphene oxide platform, the uptake of the nanomaterials is enhanced because the cell viability was affected more significantly than the pristine iron oxide nanoparticles. Little toxic effect of Fe_3_O_4_ on Cos-7 monkey kidney cells and GH3 pituitary tumor cells was observed in other analysis [60,61]. Figure 11 showed no dose-response relationship. Studies performed by Kai et al. indicated that the highest viability of BEL-7402 human hepatoma was observed when Fe_3_O_4_ nanoparticles at the concentration of 0.05 mg/mL were introduced to the cell culture [62]. When MgNPs-Fe_3_O_4_ was tested on A549 cell line for 24 h, no change in the cell viability was observed. The results of the Alamar Blue assay showed that treatment with 100 μg/mL of MgNPs-Fe_3_O_4_ for 72 h caused a significant reduction of cell viability [63].

As shown above, some difficulties in the interpretation of the obtained results can arise from variety of the factors that can influence the cell response [63]. Some of the factors are not clearly determined. Regarding the effects of graphene oxide and hybrid GO-Fe_3_O_4_ on cell viability, the mechanism is not well explained and still requires further analysis.

The *in vitro* studies play key role in exploration of the nanomaterial properties in biological environment and interaction with the living matter. The toxicity of the magnetic nanoparticles on biological entities is highly dependent on a range and combination of factors related to the properties of those nanoparticles. The physical properties such as the particle size, shape, and surface coating can evoke a toxic response by aggregating and coagulating according to size and shape. The chemical composition of the particles themselves can be naturally toxic. Here, we clearly demonstrate that the chemical functionalization of GO and Fe_3_O_4_ is a way to enhance the biocompatibility of the system and makes the system independent of the size of graphene oxide. Therefore, we believe that the obtained product with high cytocompatibility would be suitable for the application in biomedicine, e.g., as a drug carrier and/or in hyperthermia.

## Conclusions

We report a facile method of the preparation of graphene oxide-Fe_3_O_4_ nanoparticle hybrid. We prove that it is possible to increase biocompatibility of graphene oxide through the deposition of magnetite nanoparticles on the graphene oxide flakes via chemical interaction. Furthermore, we indicate that the differences in flake size do not result in different cell viability in contact with our systems. These results show the potential application of this hybrid in hyperthermia treatment. Further investigation needs to be performed in order to prove the safety and efficiency of these systems *in vivo*.

## Competing interests

The authors declare that they have no competing interests.

## Authors’ contributions

KU and MA carried out the synthesis and characterization of graphene oxide and graphene oxide-magnetite nanoparticle nanocomposites. RR carried out the synthesis of magnetite nanoparticles. MJ and MJ participated in the cytocompatibility studies and performed statistical analysis. EM and XC participated in the design of the study and coordination and helped to draft the manuscript. All authors read and approved the final manuscript.
